# Investigating the causal link between metformin and lung cancer risk: a two-sample mendelian randomization analysis

**DOI:** 10.1186/s12885-025-15494-x

**Published:** 2026-01-19

**Authors:** Yanping Feng, Yu Qiao, Huiyao Li, Bo Shen, Junli Ding, Dong Hua

**Affiliations:** 1 The Affiliated Children’s Hospital of Jiangnan University, Wuxi Medical School, Jiangnan University, Wuxi, China; 2 Department of Oncology, Wuxi Medical Center, The Affiliated Wuxi People’s Hospital of Nanjing Medical University, Wuxi, China

**Keywords:** Mendelian randomization analysis, Metformin, Lung cancer

## Abstract

Lung cancer ranks as the most prevalent malignancy worldwide. Current studies have found that metformin is associated with the occurrence of lung cancer. Nevertheless, these results are not consistent. This study seeks to explore the potential causal connection among them utilizing a two-sample Mendelian randomization (MR) approach. Genome-wide association studies (GWAS) databases were subjected to data mining in order to detect single nucleotide polymorphisms (SNPs) closely related to metformin and lung cancer, which served as instrumental variables (IVs). MR analysis was performed primarily employing the inverse variance weighted (IVW) method. The relationship between them was assessed through odds ratios (ORs). The IVW method demonstrated that there was a substantial association between metformin and a decreased risk of lung cancer (OR = 0.249, 95% CI: 0.065–0.950, *P* = 0.041) when metformin was considered as the exposure factor. The results’ credibility was confirmed by additional sensitivity analyses that showed no discernible horizontal pleiotropy or significant heterogeneity. Our study indicates that metformin may help reduce the risk of lung cancer and emphasizes the significance of its role in lung cancer prevention.

## Introduction

Lung cancer, a highly common type of malignant tumor worldwide, ranks highest in both incidence and mortality among all cancers, presenting a significant threat to human health and public safety. The latest information from the World Health Organization’s International Agency for Research on Cancer (IARC) indicates that 2.48 million new cases of lung cancer were diagnosed globally in 2020, accounting for 12.4% of all newly diagnosed cancer cases [1]. Although the exact pathogenic mechanisms of lung cancer remain incompletely understood, existing studies suggest multifactorial involvement in its development. Chronic heavy smoking and secondhand smoke exposure are identified as primary risk factors [2–4], while air pollution [5, 6], occupational exposure [7], and genetic predisposition [8] also contribute significantly. Lung cancer remains the leading cause of cancer-related mortality worldwide, despite significant advancements in elucidating the etiology of it and the advancement of precision medicine. Notably, patients with advanced-stage lung cancer have a 5-year survival rate that is as low as 5% to 15% [9]. Consequently, identifying safer and more effective preventive measures and therapies is imperative.

Diabetes mellitus is a common chronic condition among middle-aged and elderly patients, significantly increasing the risk of cardiovascular and cerebrovascular complications [10]. Studies have indicated that type 2 diabetes is associated with a higher incidence of malignant tumors, including lung [11], breast [12], colorectal [13], and pancreatic cancers [14]. Further clinical research has confirmed that diabetes also affects the survival of lung cancer patients. The coexistence of diabetes significantly shortens both progression-free survival and overall survival in these patients [15, 16]. Consequently, the improvement of glycemic control and the management of diabetes progression in patients not only reduce clinical complications but also affect the incidence and prognosis of lung cancer. The widespread use of metformin, a classical medication for type 2 diabetes derived from the natural herb French lilac, is attributed to its robust glucose-lowering effects and favorable safety profile [17, 18]. Unlike many other glucose-lowering agents, metformin does not cause clinical hypoglycemia or weight gain in patients with T2DM [19, 20], and it does not alter glucose homeostasis in non-diabetic individuals [21]. Since Evans et al. first reported in 2005 that metformin was associated with reduced tumor incidence in diabetic patients [22], researchers have increasingly focused on its potential antitumor effects [23–25]. Multiple studies have demonstrated that metformin may reduce the incidence of cancer and enhance prognosis among patients with lung cancer [26, 27], showing significant inverse correlations with the risks of gastric cancer [28], colorectal cancer [29], breast cancer [30], endometrial cancer [31], prostate cancer [32], renal cancer [33], and nasopharyngeal carcinoma [34]. A 2017 retrospective study [35] based on Taiwan’s National Health Insurance reimbursement claims database investigated a cohort of type 2 diabetes patients, including 15,414 never users and 280,159 ever users of metformin (original sample), and a 1:1 propensity score-matched cohort (*n* = 15,414 in each group, matched sample). It demonstrated a significantly lower risk of lung cancer in metformin users across both samples, with overall hazard ratios (95% CI) of 0.586 (0.509–0.674) and 0.717 (0.584–0.881). When analyzed by tertiles of cumulative duration, the highest tertile (> 46.67 months in the original sample and > 47.13 months in the matched sample) exhibited the most pronounced risk reduction, with hazard ratios of 0.176 (0.148–0.210) and 0.228 (0.146–0.357), respectively. Two additional retrospective studies [36, 37] from Taiwan, China, have also demonstrated that metformin use is associated with a reduced risk of lung cancer in patients with type 2 diabetes. Large-scale retrospective cohort studies from South Korea [38] and Lithuania [39] also confirmed a dose-dependent inverse association between them. Systematic reviews and meta-analyses by Zhang et al. [40], Wang et al. [41], Yao et al. [42] and Zhu et al. [43] further supported the evidence for metformin’s protective role in decreasing lung cancer incidence among diabetic patients. However, conflicting findings exist. For example, a study involving a cohort of 115,923 patients newly prescribed oral hypoglycemic medications observed no link between them after adjusting for confounders (including smoking) [8]. Smiechowski et al. [44] also failed to identify any link between them in a large retrospective cohort study (rate ratio = 0.94, 95% CI: 0.76–1.17), with no observed dose-response relationship for duration or cumulative dose. Similarly, a U.S. retrospective study [40] reported insufficient evidence linking metformin duration or dosage to lung cancer risk.

This study investigates the potential of metformin to lower lung cancer risk by leveraging Mendelian randomization (MR), a well-established methodological approach for causal inference in genetic epidemiology. Through the use of genetic variants as IVs, MR enables the investigation of genetically predicted causal relationships, effectively circumventing biases inherent in conventional observational study designs. The widespread application of MR in pharmacoepidemiological research and its ability to generate reliable evidence further support its validity in exploring drug-disease risk associations. By providing genetic evidence for a causal relationship between metformin and reduced lung cancer risk, this study could pave the way for repurposing metformin as a chemopreventive agent and guide precision prevention strategies in high-risk populations.

## Materials and methods

### Study design

**Fig. 1:**
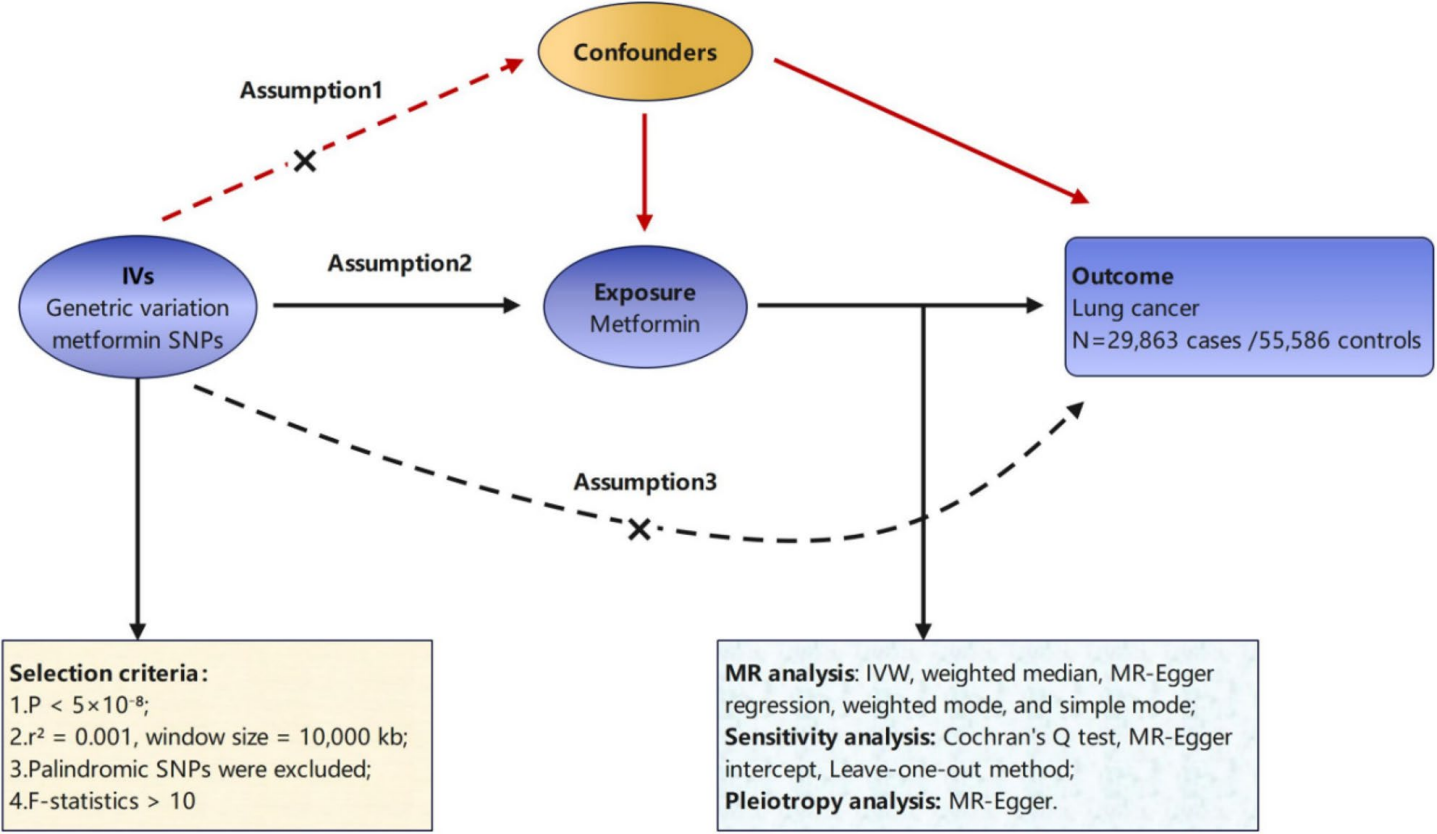
The design process and three assumptions of the MR Study

This study employed a two-sample MR methodology. The exposure was defined as metformin, with lung cancer serving as the outcome variable in the MR analysis. By leveraging genome-wide association study (GWAS) datasets pertaining to both the exposure and outcome variables, single nucleotide polymorphisms (SNPs) were selected as instrumental variables (IVs). A two-sample MR analytical framework was implemented to investigate causal relationships between metformin and lung cancer. The MR analysis adhered to three core assumptions: (1) Relevance assumption: Instrumental variables (SNPs) must be strongly associated with the exposure (metformin use) (*P* < 5 × 10⁻⁸); (2) Independence assumption: Genetic variants for the exposure must remain independent of confounders; (3) Exclusion restriction assumption: SNPs exclusively influences the outcome through the exposure, with no direct pathways.The primary analytical method was IVW, which was complemented by weighted median, MR-Egger regression, weighted mode, and simple mode for calculating ORs. Sensitivity analysis was performed using Cochran’s Q test, MR-Egger intercept and leave-one-out method to validate result reliability (Figure 1).

### Data sources

As shown in Table 1, data related to metformin and lung cancer were retrieved from publicly accessible the GWAS databases (https://gwas.mrcieu.ac.uk/). The metformin dataset (ID: ukb-a-159) from the UK Biobank database (Neale Lab) included 337,159 samples and 10,894,596 SNPs. Among them, 8,392 metformin users were defined as cases, while 328,767 non-metformin users served as controls. Metformin usage was assessed using questionnaires, medical records, and prescription data, all of which underwent rigorous quality control measures to ensure accuracy and reliability. The genetic variant data for metformin use were obtained from the UK Biobank database, The GWAS data for lung cancer were obtained from Transdisciplinary Research in Cancer of the Lung (TRICL). TRICL is a large international academic research consortium that has integrated data from eight genome-wide association studies (GWAS) on lung cancer to enhance the ability to detect genetic factors influencing all types of lung cancer. The case-control set included 29,863 lung cancer cases and 55,586 controls from a total of 85,449 samples genotyped across 10,439,018 SNPs. Datasets from European ancestry populations were selected to minimize population bias. Ethical approval and informed consent had been acquired in the initial studies; therefore, no further ethical approval was necessary for this study.

**Table 1:**
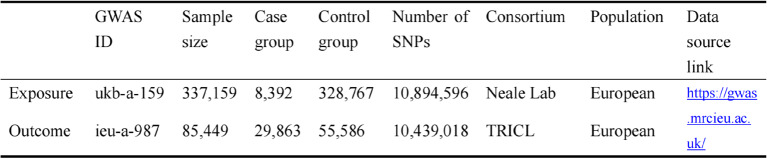
Detailed information and sources of GWAS datea for the included variables

### Instrumental variable selection

This study implemented rigorous SNP screening procedures. Initially, we extract SNPs show a notable association with metformin at the whole-genome level (*P* < 5 × 10⁻⁸). Subsequently, to ensure independence among SNPs related to the exposure factor, linkage disequilibrium (LD) pruning was performed using stringent parameters (r² = 0.001, window size = 10,000 kb). Following this, exposure-associated SNPs were extracted from the outcome dataset, and data integration was conducted by harmonizing exposure and outcome datasets through removal of incompatible alleles and all SNPs with palindromic sequences and intermediate allele frequencies. The strength of instrumental variables was evaluated using F-statistics, calculated through the formula:$\:\mathrm{F}=\left[\left(\mathrm{N}-\mathrm{K}-1\right)/\mathrm{K}\right]\times\:\left[{\mathrm{R}}^{2}/\left(1-{\mathrm{R}}^{2}\right)\right]$, where $\:{\mathrm{R}}^{2}=2\times\:\left(1-\mathrm{M}\mathrm{A}\mathrm{F}\right)\times\:\mathrm{M}\mathrm{A}\mathrm{F}\times\:{\left({\upbeta\:}/\mathrm{S}\mathrm{D}\right)}^{2}$ (N = sample size, K = number of instruments, MAF = minor allele frequency, β = effect size, SD = standard deviation). F > 10 ensures sufficient statistical power to reduce the potential bias arising from weak genetic instruments (Table 2).

**Table 2:**
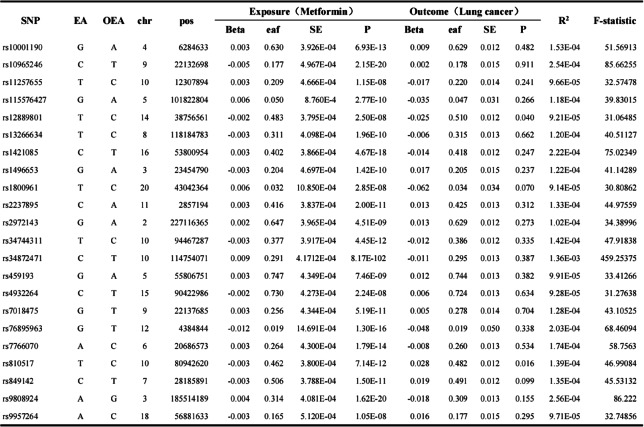
Detailed information and intensity evaluation of SNPs were included in this study

### Statistical analysis

The causal effects were estimated using the IVW method as the primary approach, which provides unbiased estimates when the instrumental variables are valid and pleiotropy is minimal. Sensitivity analyses included Cochran’s Q test for heterogeneity, MR-Egger intercept for pleiotropy, and leave-one-out analysis to identify influential SNPs. Funnel plots were generated to assess symmetry and potential pleiotropy. Results were reported as ORs with 95% CIs; *P* < 0.05 indicated statistical significance. OR < 1 suggested a protective effect of metformin, while OR > 1 indicated risk. The analyses were carried out using the Two Sample MR package in R Studio (version 4.3.3) Table 3.

**Table 3:**
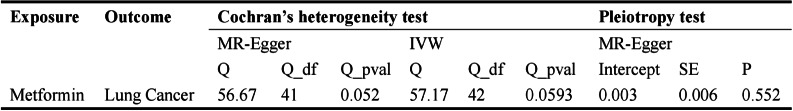
Heterogeneity and Pleiotropy analyses

## Results

### MR analysis results

After excluding linkage disequilibrium (LD) and weak instrumental variables, we selected 22 SNPS as instrumental variables for the MR analysis. All SNPs exhibited F > 10, confirming the strength of instrumental variables and ensuring sufficient statistical power for the analysis.

**Fig. 2:**
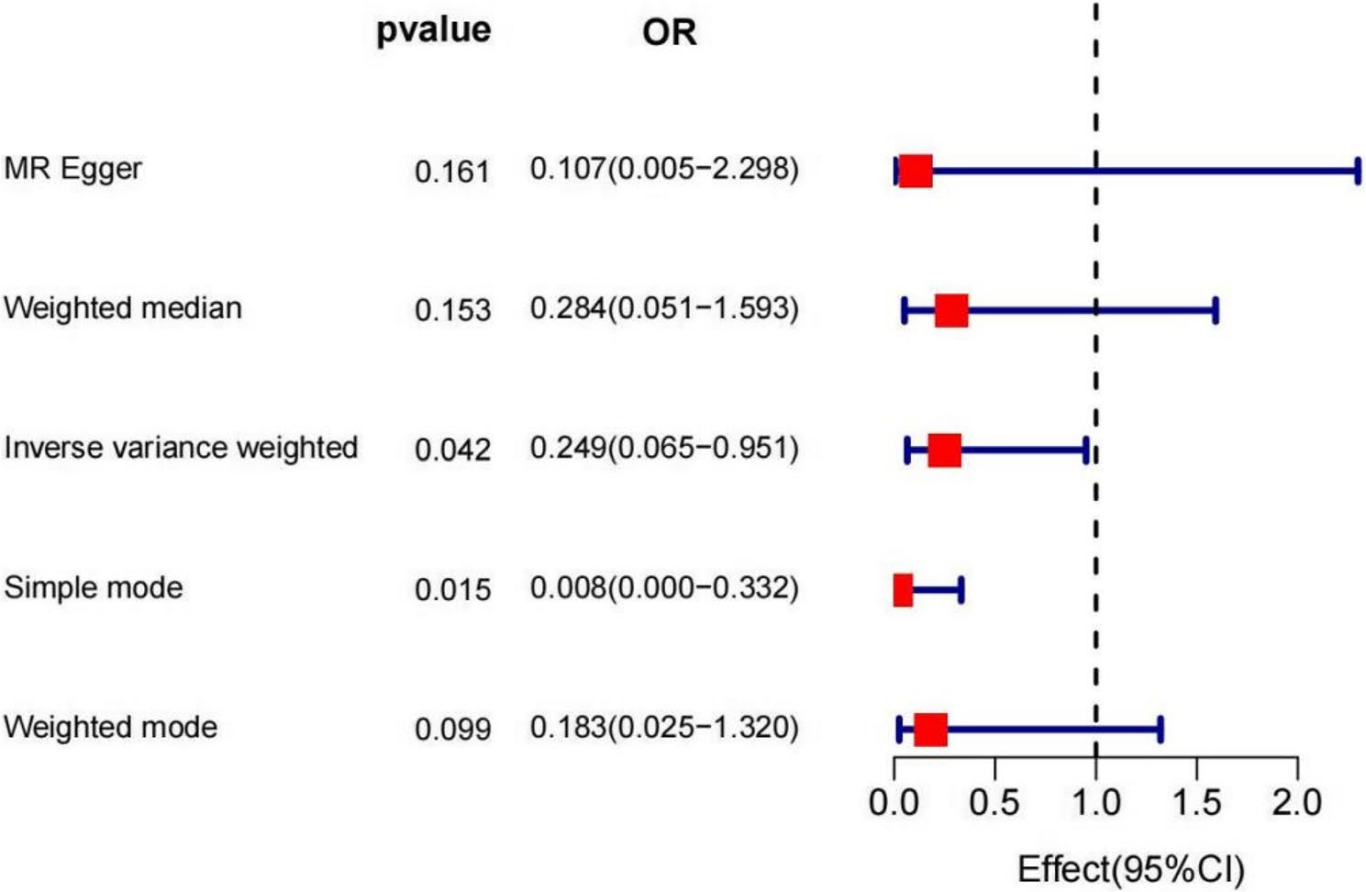
MR analysis of the association between Metformin and risk of lung cancer

**Fig. 3:**
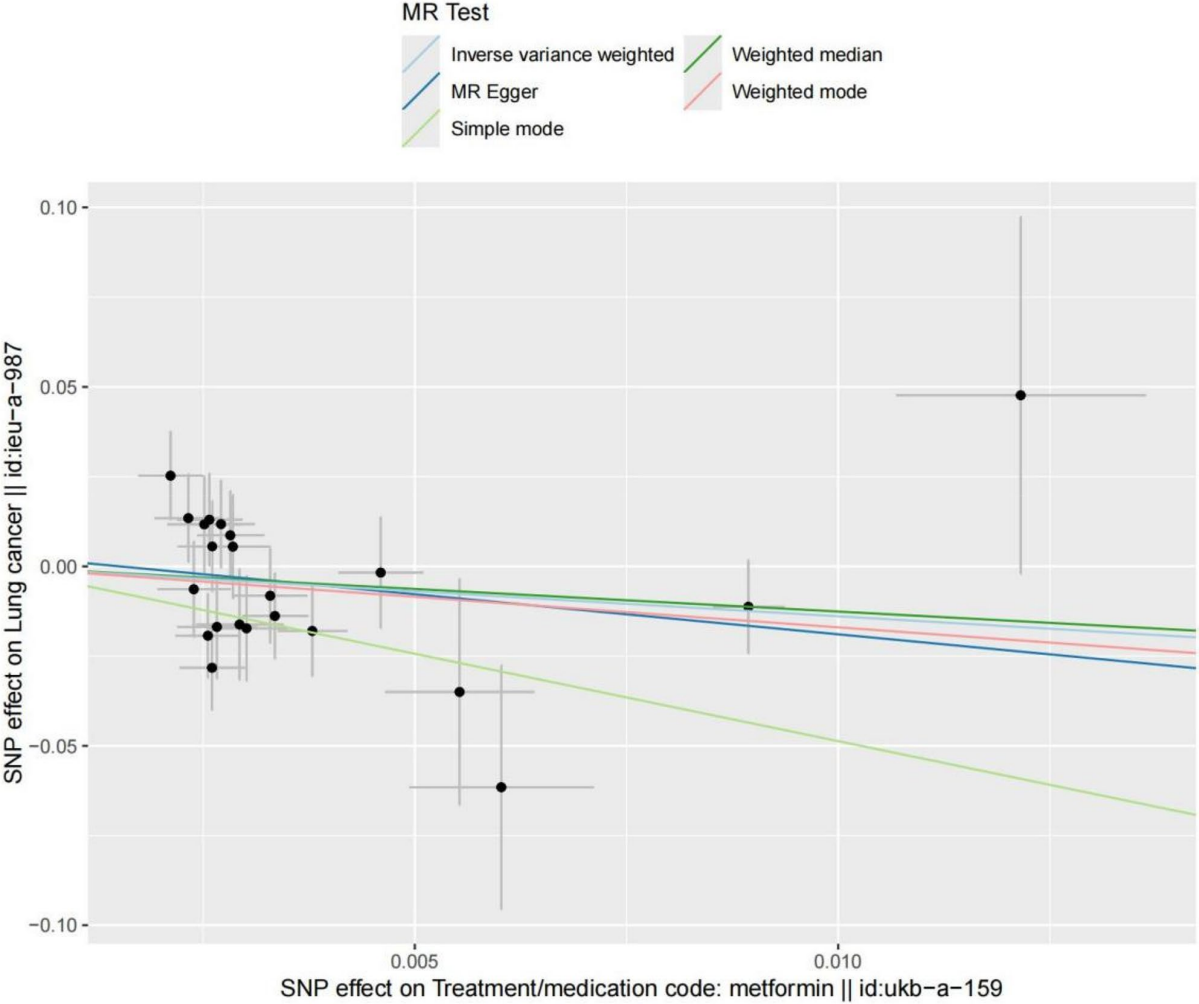
MR test scatter plot of the five methods

**Fig. 4:**
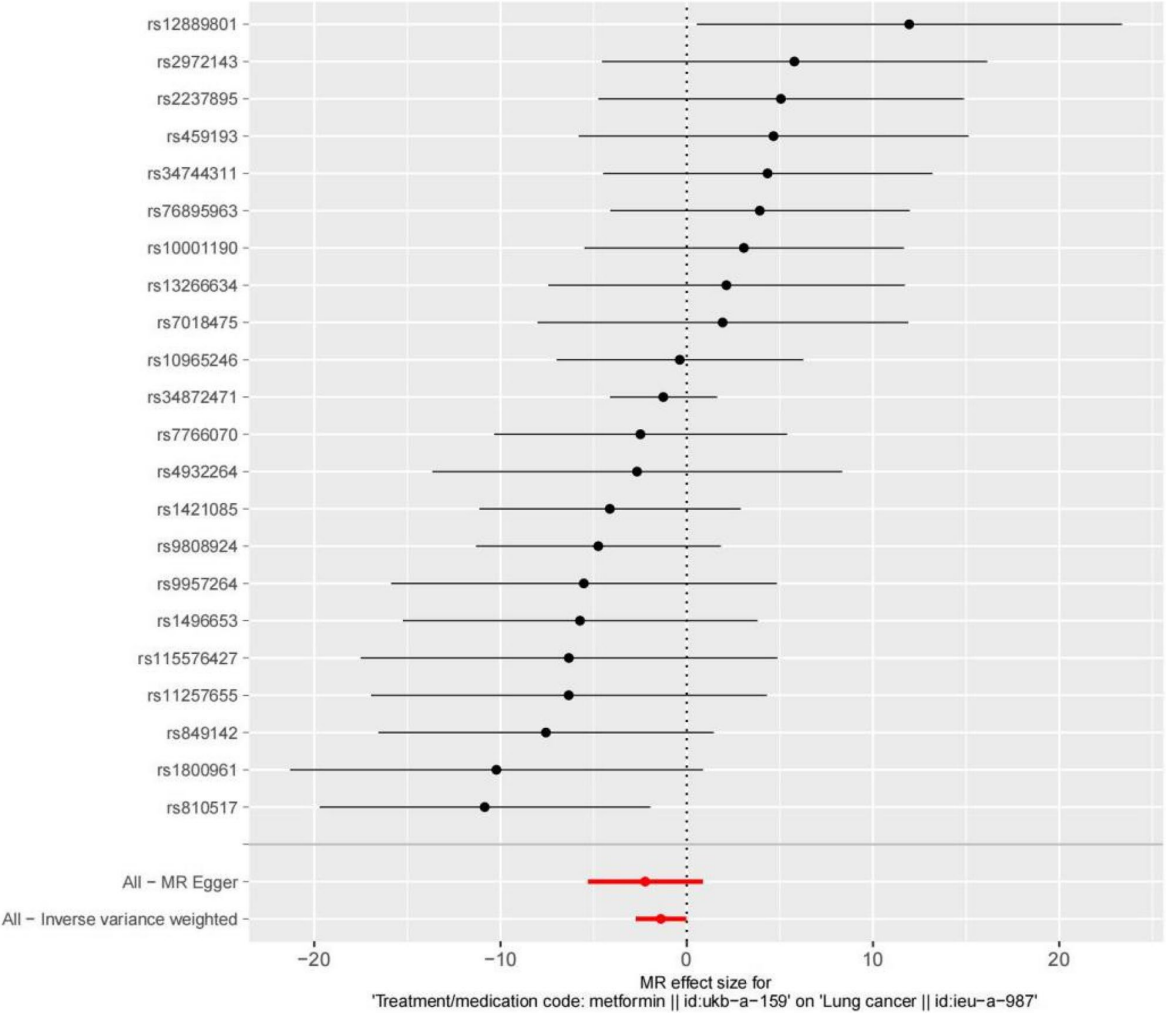
Forest plot for each SNP

As shown in Fig. 2, the β values from all five methods were negative, and the OR values were all less than 1, suggesting that metformin is a protective factor against lung cancer. Moreover, the *P*-values for the IVW and Simple mode methods were both less than 0.05, indicating statistical significance. These findings were visualized using a forest plot and a scatter plot. The forest plot demonstrated that the combined OR values in the Simple mode and IVW methods were both less than 0, while the slopes of all five methods in the scatter plot were negative, further supporting the conclusion that metformin serves as a protective factor against lung cancer (Figs. 3 and 4).

### Heterogeneity test

The fundamental principle of the MR heterogeneity test is grounded in Mendel’s law of inheritance, which states that certain biological traits are determined by genes that are randomly assigned to the next generation. By leveraging this principle, the data is randomized, and the distribution before and after randomization is compared to assess the presence of heterogeneity.

**Fig. 5:**
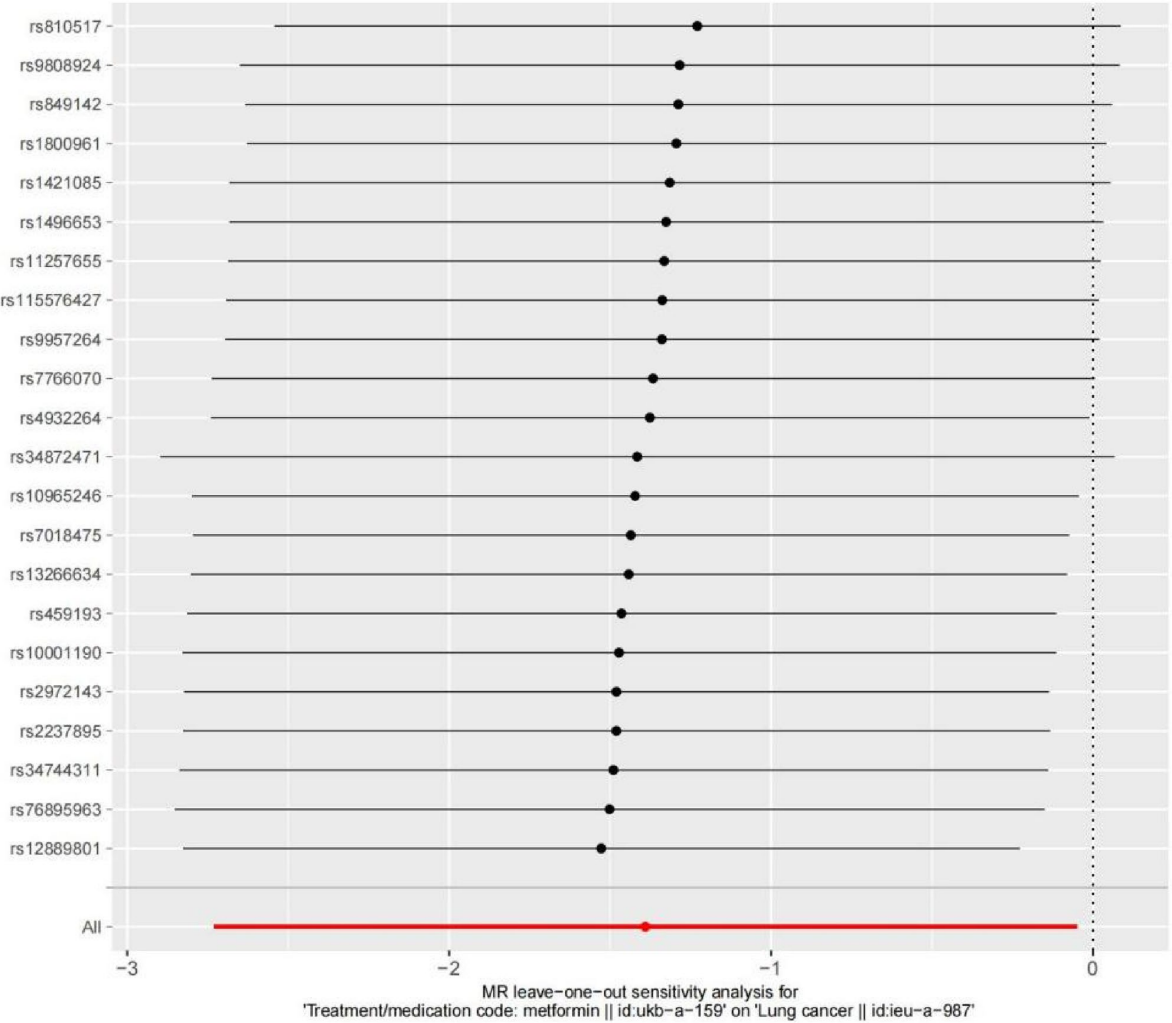
Leave-one-out analyse

In this study, the MR-Egger and IVW methods were utilized to test for heterogeneity. The results indicated that *P* > 0.05, suggesting no heterogeneity present in the data (Table 2). The leave-one-off method also demonstrated the absence of significant hetero-geneity among single-SNPs (Fig. 5).

### Pleiotropy test

The MR-Egger method was used to identify and adjust for potential outliers, and pleiotropy was eliminated by excluding these outliers. The intercept was used as a reference: if the intercept tended toward zero, the results were considered similar to those obtained using the IVW method. However, if the intercept deviated significantly from zero, it suggested a high likelihood of horizontal pleiotropy among SNPs. The pleiotropy test results showed Egger-intercept < 0.05 and *P* > 0.05 (Egger-intercept = −0.003, *P* = 0.552), indicating no presence of pleiotropy in the data (Table 2).

**Fig. 6:**
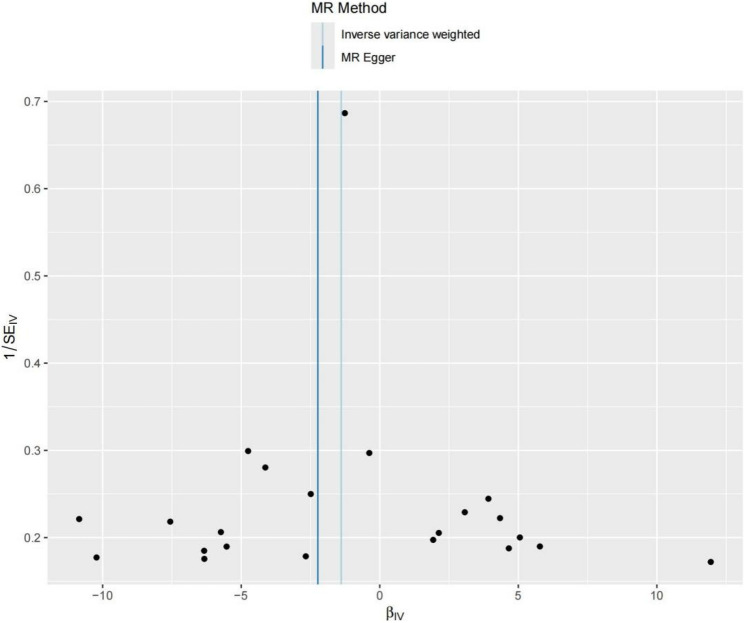
Funnel plot

Additionally, the funnel plots from both the MR-Egger and IVW methods showed that the SNPs were symmetrically distributed on both sides of the effect size reference line (vertical line), further confirming the absence of pleiotropy (Fig. 6).

### Stability assessment

Leave-one-out sensitivity analysis is a crucial step in MR analysis, primarily assess the reliability of MR findings, identify outlier SNPs, and reduce pleiotropy bias.The results indicated that after sequentially removing individual SNPs and re-conducting the MR analysis, the effect estimates remained consistent, with all confidence interval error bars predominantly located to the left of zero. No single SNP was found to have a significant impact on the causal effect estimation, demonstrating that the evaluation of the causal relationship was not driven by any single genetic instrument. This confirms the reliability and robustness of the results, further supporting the stability of the MR analysis in this study (Fig. 5).

## Discussion

This study utilized two-sample MR analysis to examine the causal relationship between metformin and lung cancer risk. Our findings demonstrated that metformin was significantly linked to a decreased risk of lung cancer incidence in individuals of European ancestry (OR = 0.249, 95% CI: 0.065–0.950, *P* = 0.041). Sensitivity analyses further confirmed the robustness of these results.

The study conducted in Taiwan reported a hazard ratio of 0.228 (95% CI: 1.460 − 0.357) for the highest tertile of cumulative metformin exposure (> 47.13 months) in the matched cohort, which aligns closely with the hazard ratio of 0.249 (95% CI: 0.065–0.950) derived from our MR analysis. While the retrospective study captured long-term medication effects through extended follow-up, our MR study evaluated the lifelong cumulative effect of metformin exposure. This consistency suggests that the potential anticancer effect of metformin may require long-term accumulation, and the risk of lung cancer may gradually decrease with prolonged exposure. Other clinical retrospective studies [36, 45–47] also directly support the “long-term accumulation” hypothesis. Furthermore, a review [48] delves into the mechanisms by which metformin targets cancer stem cells through mTOR pathway inhibition and AMPK activation. The eradication of cancer stem cells is crucial for preventing tumor recurrence and achieving long-term disease control, but this process is inherently gradual and requires sustained pharmacological exposure to manifest therapeutic effects. The consistency of findings across these methodologically distinct studies reduces the likelihood that the observed association between metformin and lung cancer risk is due to confounding or bias, thereby lending robust support to the hypothesis that metformin may causally reduce the risk of lung cancer. Future prospective clinical trials are warranted to definitively establish its clinical utility.

Despite many clinical studies indicating that metformin might have antitumor effects, its underlying mechanisms remain incompletely understood. Research has demonstrated that metformin exerts antitumor activity through multiple interconnected pathways. It activates AMPK by inhibiting mitochondrial complex I and reducing ATP levels, leading to downstream suppression of mTOR signaling [49–52] and activation of the p53/p21 axis to inhibit cancer cell proliferation [53]. Metformin also ameliorates hyperglycemia and insulin resistance to reduce IGF-1 levels [54], with the Memmott team [55] confirming in mouse models that metformin suppresses lung tumorigenesis through IGF-1R/IR and AKT-mediated mTOR inhibition.In terms of metastasis suppression, it blocks STAT3 phosphorylation to inhibit interleukin-6-induced epithelial-mesenchymal transition and tumor metastasis [56]. Furthermore, a study published in 2023 [57] reported that metformin triggers ferroptosis in A549 and H1299 lung cancer cells via the Nrf2/HO-1 signaling pathway, thereby enhancing its antitumor activities. These elaborated mechanisms not only clarify metformin’s role in lung cancer intervention but also show promising implications for lung cancer therapeutics.

This investigation employs MR method to establish genetic-based causal associations between metformin exposure and reduced lung cancer risk. In this method, the genetic variation is randomly distributed across the population, minimizing confounding biases prevalent in conventional observational studies.The study demonstrates methodological advancements in overcoming inherent limitations of observational research paradigms, thereby proposing novel conceptual frameworks for subsequent investigations into chronic disease interventions.

However, this study has several limitations. First, the GWAS data used in this study were derived from European populations; therefore, the extrapolation of our findings to other ethnic groups should be cautiously considered. While the aforementioned retrospective studies demonstrated an association between metformin and lung cancer risk in Chinese [35–37], Korean [38], and Lithuanian [39] populations, the conclusions from our analysis do not possess universal applicability. Second, metformin is often used in combination with other glucose-lowering medications, and our analysis did not account for potential drug-drug interactions. Previous studies have suggested that other antidiabetic drugs, such as TZD [58, 59], acarbose [60], DPP4i [61], SGLT2i [62, 63], GLP-1RA [64, 65] etc. as well as medications for diabetic complications like beta-blockers and statins [66–68], aspirin [69, 70], ACEIs/ARB [71, 72] etc. may also reduce lung cancer risk. These factors could introduce potential confounding effects and biases in our study. Finally, although we employed MR-Egger regression to address horizontal pleiotropy, selected strong genetic instruments (all F-statistics > 10) to minimize weak instrument bias, and utilized a two-sample MR design with summary statistics from different consortia to reduce sample overlap, the current GWAS databases still lack stratification of samples with varying disease severity. More detailed clinical data remain incomplete. Potential confounding factors (e.g., insulin resistance and metabolic syndrome [73, 74], smoking [2–4], air pollution [5, 6] and environmental and occupational exposures [7]) as well as potential inaccuracies in the exposure proxy may affect the results.

## Conclusion

In summary, this study suggests a potential association between metformin use and reduced lung cancer risk, which may hold significant implications for future prevention and treatment strategies. Accumulating evidence from Clinical observation, basic research, and our analysis consistently underscores metformin’s potential role in lung cancer. Future research warrants further prospective studies and deeper mechanistic investigations (such as utilizing scRNA-seq on relevant models or patient samples) to elucidate the specific mechanisms through which metformin may exert its protective effects.

## Data Availability

The datasets generated and analyzed during the current study are available in the GWAS summary data repository at https://gwas.mrcieu.ac.uk/.

## Abbreviations

Abbreviations: Definion
MR: Mendelian Randomization
GWAS: Genome-wide Association Study
SNP: Single Nucleotide Polymorphism
IV: Instrumental Variable
IVW: Inverse Variance Weighted
OR: Odds Ratio
IARC: International Agency for Research on Cancer
LD: Linkage Disequilibrium
AMPK: Adenosine Monophosphate-activated Protein Kinase
mTOR: Mammalian Target of Rapamycin
NSCLC: Non-small Cell Lung Cancer
EMT: Epithelial-mesenchymal Transition
